# Inter-hospital transfer for mechanical thrombectomy within the supraregional stroke network NEVAS

**DOI:** 10.1007/s00415-020-10165-2

**Published:** 2020-09-05

**Authors:** Katharina Feil, Jan Rémi, Clemens Küpper, Moriz Herzberg, Franziska Dorn, Wolfgang G. Kunz, Paul Reidler, Johannes Levin, Katrin Hüttemann, Steffen Tiedt, Wanja Heidger, Katharina Müller, Dennis C. Thunstedt, Rainer Dabitz, Robert Müller, Thomas Pfefferkorn, Gerhard F. Hamann, Thomas Liebig, Marianne Dieterich, Lars Kellert

**Affiliations:** 1 Department of Neurology, Ludwig Maximilians University (LMU), Marchioninistrasse 15, 81377 Munich, Germany; 2 German Center for Vertigo and Balance Disorders, Ludwig Maximilian University (LMU), Munich, Germany; 3 Institute of Neuroradiology, Ludwig Maximilian University (LMU), Munich, Germany; 4 Department of Radiology, Ludwig Maximilian University (LMU), Munich, Germany; 5 German Center for Neurodegenerative Diseases (DZNE), Munich, Germany; 6 Munich Cluster for Systems Neurology (SyNergy), Munich, Germany; 7 Institute for Stroke and Dementia Research, Ludwig Maximilian University (LMU), Munich, Germany; 8 Department of Neurology, Klinikum Ingolstadt, Ingolstadt, Germany; 9 Department of Neurology and Neurological Rehabilitation, Bezirkskrankenhaus Günzburg, Günzburg, Germany

**Keywords:** Stroke, Mechanical thrombectomy, Endovascular therapy, Outcome, Thrombolytic therapy, t-PA, Reperfusion, Transportation, Drip and ship, Telemedicine stroke network

## Abstract

**Background:**

Telemedicine stroke networks are mandatory to provide inter-hospital transfer for mechanical thrombectomy (MT). However, studies on patient selection in daily practice are sparse.

**Methods:**

Here, we analyzed consecutive patients from 01/2014 to 12/2018 within the supraregional stroke network “Neurovascular Network of Southwest Bavaria” (NEVAS) in terms of diagnoses after consultation, inter-hospital transfer and predictors for performing MT. Degree of disability was rated by the modified Rankin Scale (mRS), good outcome was defined as mRS ≤ 2. Successful reperfusion was assumed when the modified thrombolysis in cerebral infarction (mTICI) was 2b-3.

**Results:**

Of 5722 telemedicine consultations, in 14.1% inter-hospital transfer was performed, mostly because of large vessel occlusion (LVO) stroke. A total of *n* = 350 patients with LVO were shipped via NEVAS to our center for MT. While *n* = 52 recanalized spontaneously, MT-treatment was performed in *n* = 178 patients. MT-treated patients had more severe strokes according to the median National institute of health stroke scale (NIHSS) (16 vs. 13, *p* < 0.001), were more often treated with intravenous thrombolysis (64.5% vs. 51.7%, *p* = 0.026) and arrived significantly earlier in our center (184.5 versus 228.0 min, *p* < 0.001). Good outcome (27.5% vs. 30.8%, *p* = 0.35) and mortality (32.6% versus 23.5%, *p* = 0.79) were comparable in MT-treated versus no-MT-treated patients. In patients with middle cerebral artery occlusion in the M1 segment or carotid artery occlusion good outcome was twice as often in the MT-group (21.8% vs. 12.8%, *p* = 0.184). Independent predictors for performing MT were higher NIHSS (OR 1.096), higher ASPECTS (OR 1.28), and earlier time window (OR 0.99).

**Conclusion:**

Within a telemedicine network stroke care can successfully be organized as only a minority of patients has to be transferred. Our data provide clinical evidence that all MT-eligible patients should be shipped with the fastest transportation modality as possible.

## Introduction

In general, telemedicine stroke networks provide neurological expertise to hospitals with limited access to direct neurological and neurointerventionalist care. The major questions in telemedicine stroke consultations are clinical decisions about treatment with intravenous thrombolysis (IVT) and whether the individual patient has to be transferred to a comprehensive stroke center where mechanical thrombectomy (MT) can be performed. Not all transferred patients actually undergo MT. Data on patients who were transferred for MT via drip and ship, but finally did not receive endovascular treatment for different reasons are sparse [1–3]. However, since 2015 MT in combination with IVT became standard of care in patients with ischemic stroke due to large vessel occlusion (LVO) [4]. Providing MT for all eligible patients is currently a challenging and important task in acute stroke care including telemedicine stroke networks. In this study we focus on patients with proven LVO that were transferred for MT within our supraregional stroke network NEVAS (NEuroVAskulaeres Netzwerk Suedwestbayern) [5] with the intention to perform MT but irrespective whether MT was finally performed or not to identify underlying decision-making processes in these patients. Thus, we compared clinical, radiological and outcome parameters of MT-treated versus not treated patients who were shipped via drip and ship with LVO in real-life stroke care within the supraregional telemedicine stroke network NEVAS.

## Methods

### Description of the supraregional stroke network

All consecutive patients between January 2014 and December 2018 who were shipped to our comprehensive stroke center via the supraregional stroke network “Neurovascular Network of Southwest Bavaria” (Neurovaskulaeres Netzwerk Suedwestbayern, NEVAS) were analyzed. For a detailed description of the stroke care organization within this network see reference [5]. In the different regional hospitals where the patients initially presented with suspected stroke, a telemedicine consultation was made within NEVAS via video stream and digital DICOM-cloud-transfer of radiological imaging and clinical advice for further procedures and treatment as well as the decision about secondary transportation was given by experienced stroke specialists (for further details see reference [5]). The decision for inter-hospital transfer within NEVAS was based on clinical as well as imaging findings.

### Study population

We retrospectively analyzed prospectively collected data including demographic parameters, vascular risk factors, periprocedural time intervals, treatment complications, and data on clinical outcome. Stroke severity was assessed using the National Institute of Health Stroke Scale (NIHSS). Degree of dependence or disability was rated by the modified Rankin Scale (mRS) and the premorbid mRS (pmRS) respectively. Signs of early ischemic damage on non-contrast computed tomography (CT) were recorded according to the Alberta Stroke Program Early CT Score (ASPECTS).

Site of LVO was defined based on CT-angiography or angiograms prior MT. An independent neuroradiologist rated recanalization success based on final angiograms according to the modified Thrombolysis in Cerebral Infarction (mTICI) score in the anterior circulation. Successful reperfusion was defined as mTICI 2b-3 [6].

According to recent clinical trials, we defined absolute indication for MT for patients with the site of occlusion in the M1-Segment of the middle cerebral artery (MCA), in the internal carotid artery (ICA) as well as in the carotid-T. Intracerebral hemorrhages (ICH) were defined according to ECASS-3 [7] (any hemorrhage with neurologic deterioration as indicated by a NIHSS that was 4 points higher than the value at baseline or the lowest value in the first 7 d or any hemorrhage leading to death; in addition, the hemorrhage must have been identified as the predominant cause of the neurological deterioration). Clinical outcome was assessed either by phone calls or during outpatient follow-up visits. Good outcome was defined as mRS of 0–2.

### Statistical analysis

Continuous variables were tested for normal distribution using the Kolmogorov–Smirnov test. For normally distributed data the results were presented as mean and standard deviation (± SD); for non-normally distributed data as median (25%, 75% percentile) and interquartile range (IQR), or counts and percentages. Baseline characteristics, periprocedural time intervals, and outcome parameters were compared in the different groups using the *T* Test, the Mann–Whitney *U* test, Kruskal–Wallis *H* or the Chi-square test, where appropriate. Differences were considered significant if *p* value was < 0.05. Logistic regression analysis with the good outcome at 3 months as dependent variable or MT as dependent variable respectively were calculated using significant parameters from the univariate analysis as well as well-known predictors. For logistic regression analysis categorical variables were defined as follows: sex male = 0, female = 1, IVT treatment no = 0, yes = 1, successful recanalization no = 0, yes = 1, onset unknown no = 0, yes = 1; sICH ECASS 4 no = 0, yes = 1; complications no = 0; yes = 1; ground transportation no = 0, yes = 1. Data were collected and evaluated using Excel (Microsoft, Redmond, WA) spreadsheet software. Statistical analysis was performed using SPSS (IBM, Armonk, NY).

### Ethics statement

This study was approved by the local ethics committee (project number 17–174) and was performed in accordance with the ethical standards laid down in the 1964 Declaration of Helsinki and its later amendments.

### Data availability

The data that support the findings of this study are available on request from the corresponding author. The data are not publicly available due to privacy or ethical restrictions.

## Results

### Patient characteristics

**Fig. 1:**
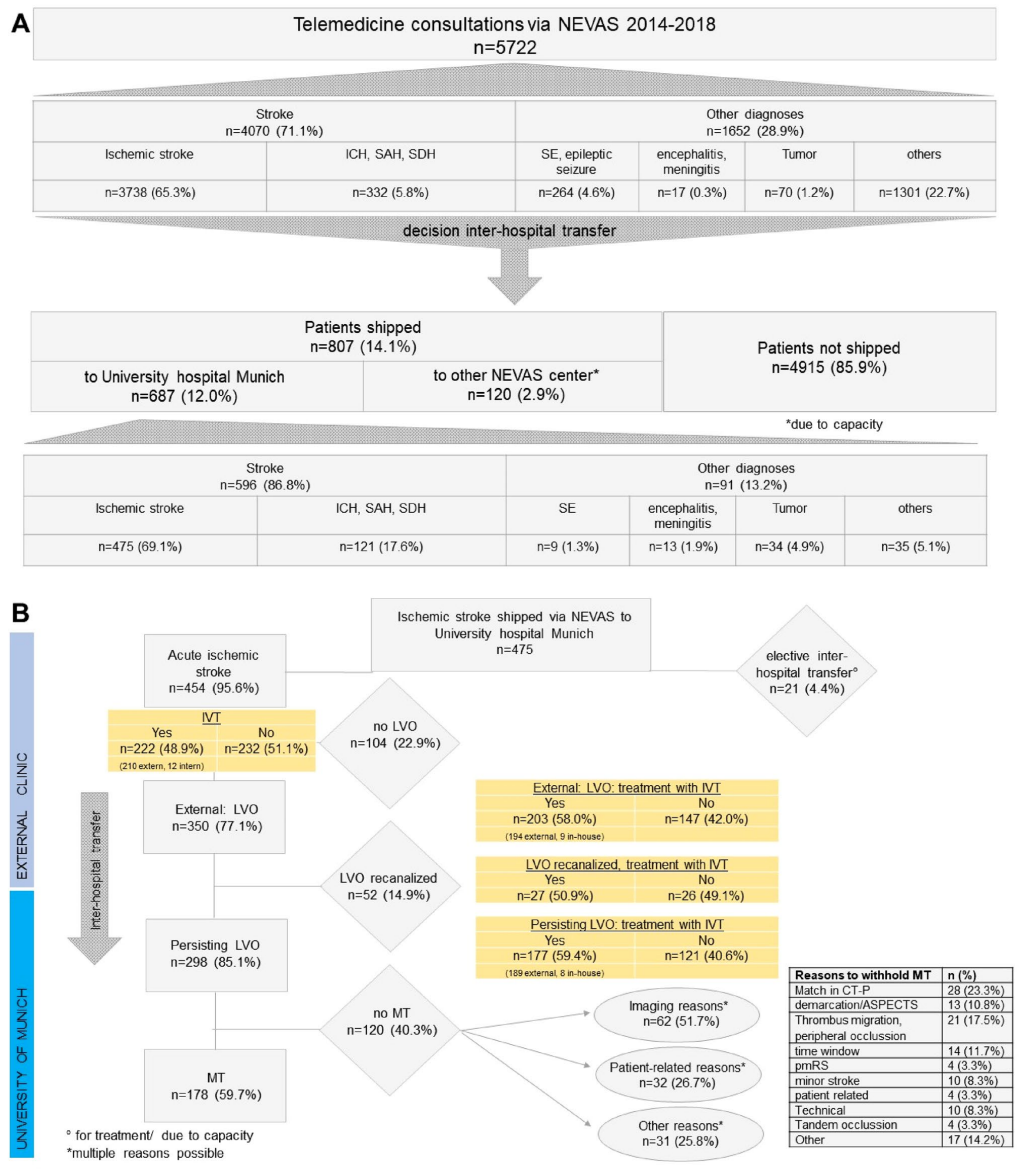
A Flow chart of all telemedicine consultations via NEVAS and diagnosis of these patients before and after the decision for inter-hospital transfer. B: Flow chart of acute ischemic stroke patients with or without large vessel occlusion (LVO) and treatment options. *ASPECTS* Alberta Stroke Program Early CT Score, *CT-P* computed tomography perfusion, *ICH* intracerebral hemorrhage, *IVT* intravenous thrombolysis, *LVO* large vessel occlusion, *MT* mechanical thrombectomy, *n* number, *NEVAS* Neurovascular Network of Southwest Bavaria, *pmRS* premorbid modified Rankin Scale, *SAH* subarachnoidal hemorrhage, *SE* status epilepticus, *SDH* subdural hemorrhage

During the study period between 2014 to 2018, 5722 patients underwent telemedicine consultation within NEVAS. Figure 1a distributes the diagnoses of these patients: 71.1% were ischemic strokes or transient ischemic attacks (TIA) while intracranial hemorrhage (ICH), subarachnoid hemorrhages (SAH) and subdural hemorrhages (SDH) occurred in *n* = 332 (5.8%) patients (see Fig. 1a).

Decision for inter-hospital transfer from different primary regional hospitals in rural areas after telemedicine consultation via NEVAS was made in a total of *n* = 807 patients (14.1%). Due to capacity and network organization, a total of *n* = 687 (12.0%) were shipped to our comprehensive stroke center at the University of Munich. Of these patients who were shipped to our center, *n* = 475 patient suffered from ischemic stroke and *n* = 212 were transferred because of other diagnoses (see Fig. 1b). Out of *n* = 350 patients presenting with ischemic stroke due to LVO, *n* = 52 (14.9%) had already recanalized LVO during inter-hospital transfer and before MT. Out of the remaining *n* = 298 patients with persisting LVO, *n* = 178 underwent MT while in *n* = 120 MT was withheld. Main reasons for withholding MT were either based on imaging data (*n* = 62, 51.7%) with no relevant mismatch in CT-perfusion (*n* = 28, 23%), thrombus migration with no proximal LVO (*n* = 21, 17.5%), infarct demarcation/lower ASPECTS (*n* = 13, 10.8%) or patient-related data with clinical improvement or minor stroke (*n* = 20, 16.6%) poor premorbid status (*n* = 4, 3.3%) or prolonged-time window (*n* = 14, 11.7%) (for details see Fig. 1b). Table 1 shows baseline characteristics of acute stroke patients with recanalized LVO and patients with persisting LVO in comparison of patients treated with MT vs. not treated patients.

**Table 1:** Baseline data and clinical characteristics of acute stroke patients with persisting LVO comparing the MT-group vs. the no-MT-group patients (*n* = 298) and recanalized LVO (*n* = 52)

	Acute stroke patients with recanalized LVO after drip and ship (*n* = 52)		Acute stroke patients with persisting LVO after drip and ship (*n* = 298)		
		*p* value^a^	MT *n* = 178 (59.7%)	No MT *n* = 120 (40.3%)	*p* value
Age—years (SD)	71.2 ± 14.5	0.875	71.5 ± 13.3	70.0 ± 14.1	0.357
Sex—female, *n* (%)	18 (34.6%)	0.438	81 (45.5%)	39 (32.5%)	0.025
Risk factors					
Arterial hypertension, *n* (%)	40 (76.9%)	0.315	130 (73.0%)	82 (68.3%)	0.302
Diabetes mellitus, *n* (%)	9 /17.3%)	0.752	29 (16.3%)	18 (15.0%)	0.734
Hypercholesterolemia, *n* (%)	15 (28.8%)	0.232	41 (23.0%)	23 (19.2%)	0.440
Smoking, *n* (%)	15 (28.8%)	0.473	34 (19.1%)	29 (24.2%)	0.588
AF, *n* (%)	13 (25.0%)	0.006	82 (46.6%)	53 (44.2%)	0.691
CHA2DS2-VASC-Score, median (IQR)	5 (4, 6)	0.455	5 (4, 6)	5 (4, 6)	0.300
Clinical characteristics					
pmRS, median (IQR)	0 (0, 1)	0.151	0 (0, 1)	0 (0, 1)	0.668
pmRS ≤ 2	48 (92.4%)		160 (89.9%)	112 (93.3%)	
Baseline NIHSS at center, median (IQR)	4 (1, 9)	< 0.0001	16 (11, 21)	13 (4, 20)	< 0.0001
Imaging data					
ASPECTS, median (IQR)	10 (9, 10) (*n* = 29)	0.003	8 (7, 10) (*n* = 107)	8 (6, 10) (*n* = 83)	0.041
Site of symptomatic LVO^a^, *n* (%)					0.063
Intern LVO	0 (0%)		178 (100%)	120 (100%)	
ICA, extracranial			18	39	
ICA, intracranial			12	18	
Carotid-T			20	8	
MCA, M1			115	41	
MCA, M2			16	22	
Vertebral artery			7	10	
Basilar artery			22	8	
Tandem lesion			32 (18.0%)	29 (24.2%)	0.195
Time intervals					
Onset to IVT—minutes, median (IQR)	117.5 (93.3, 146.3)	0.002	100.0 (73.8, 135.0)	100.0 (75.0, 145.0)	0.373
Adjusted transportation time^b^—minutes, median (IQR)	103.0 (75.0, 113.5) (*n* = 17)	0.771	86.5 (62.0, 114.8) (*n* = 104)	101.0 (80.0, 146.0) (*n* = 55)	0.168
Symptom onset to door at our center—minutes, median (IQR)	278.0 (204.5, 1281.5)	0.955	184.5 (141.8, 243.5)	228.0 (183.3, 399.5)	< 0.001
Treatment					
IVT, *n* (%)	26 (50.0%)	0.208	115 (64.6%)	62 (51.7%)	0.026
Absolute indication for MT			124 (69.7%)	47 (39.2%)	< 0.001
mTICI 2b-3, *n* (%)			131 (73.6%)		

*AF* atrial fibrillation, *ASPECTS* Alberta Stroke Program Early CT Score, *ICA* intracranial carotid artery, *IQR* interquartile range, *IVT* intravenous thrombolysis, *MCA* Middle cerebral artery, *mTICI* modified thrombolysis in cerebral infarction, *n* number

^a^Multiple occlusion sites possible

^b^In patients with IVT in the external clinic (adjusted time for transportation from time of IVT to door in our stroke center)

In cases with persisting LVO, MT-treated patients were more like to be female (45.5% vs. 32.5%, *p* = 0.025) and were transferred more by airborne transport (34.8% vs. 15.0%, *p* = 0.011). They were more often treated with IVT (64.5% vs. 51.7%, *p* = 0.026), and had more severe strokes according to the NIHSS (16 vs. 13, *p* < 0.001). With respect to time intervals MT-treated patients arrived significantly earlier in the comprehensive stroke center (184.5 vs. 228.0, *p* < 0.001). Median time from stroke symptom onset to arrival at our center was 205.0 min in airborne transfer versus 228.0 min in-ground transfer (*p* = 0.010). However, in patients with recanalized LVO median time was 278.0 min but did not differ significantly from patients with persisting LVO (*p* = 0.955). Adjusted transportation time (in patients with IVT in the external clinic; time from IVT to arrival at the door in stroke center, in *n* = 176) did not differ significantly comparing patients with recanalized LVO (103.0 min) to patients with persisting LVO (*p* = 0.771) who were treated with MT (86.5 min) or not (101.0 min, *p* = 0.168). However, adjusted median transportation time differed significantly comparing airborne transfer and ground transfer (102.0 min, IQR 83.0–121.0 versus 85.0 min, IQR 61–121, *p* = 0.004).

### Complications and outcome

Overall complications were more frequent in treated vs. not-treated patients (33.7% vs. 18.3%, *p* = 0.003) (for details see Table 2). The rate of sICH was 6.2% in the MT-treated group and 3.3% in the no-MT-group (*p* = 0.58). At 3 months good functional outcome (27.5% vs. 30.8%, *p* = 0.35) and mortality (32.6% vs. 23.5%, *p* = 0.79) was comparable in both groups (for details see Table 3).

**Table 2:** Complications of acute stroke with persisting LVO according to MT-group vs. no-MT-group patients (*n* = 298) and recanalized LVO (*n* = 52)

	Acute stroke patients with recanalized LVO after drip and ship (*n* = 52)		Acute stroke patients with persisting LVO after drip and ship (*n* = 298)		
		*p* value^a^	MT *n* = 178 (59.7%)	No MT *n* = 120 (40.3%)	*p* value
sICH ECASS 4, *n* (%)	1 (1.9%)	0.577	11 (6.2%)	4 (3.3%)	0.581
Complications during hospital stay, *n* (%)	1 (1.9%)	< 0.001	60 (33.7%)	22 (18.3%)	0.003
Malignant MCA infarction, *n* (%)	0	0.017	15 (8.4%)	15 (12.5%)	0.253
Periprocedural complications, *n* (%)			16 (9.0%)		
Groin hematoma			3		
Vasospasm			8		
Technical			3		
Dissection			2		

*MCA* middle cerebral artery, *n* number, *LVO* large vessel occlusion, *sICH* symptomatic intracranial haemorrhage

^a^Comparing the group of patients with recanalized LVO to the group of patients with persisting LVO

**Table 3:** Outcome of acute stroke patients with persisting LVO comparing the MT-group vs. the non-MT-group patients (*n* = 298) and recanalized LVO (*n* = 52) at 3-month follow-up (available in *n* = 267 patients, 89.6%)

	Acute stroke patients with recanalized LVO after drip and ship (*n* = 52)		Acute stroke patients with persisting LVO after drip and ship (*n* = 298)		
		*p* value^a^	MT *n* = 178 (59.7%)	No MT *n* = 120 (40.3%)	*p* value
3-months outcome	Available in *n* = 41 patients		Available in *n* = 163 (91.6%)	*n* = 104 (86.7%)	
mRS, median, (IQR)	2 (0, 5)	< 0.001	4 (2, 6)	5 (2, 6)	0.786
Good functional outcome (mRS 0–2) *n* (%)	22 (42.3%)	0.007	49 (27.5%)	36 (30.0%)	0.349
Mortality, *n* (%)	6 (11.5%)	0.006	58 (32.6%)	39 (32.5%)	0.752
Good outcome in patients with absolute indication, *n* (%)			27 (21.8%)	6 (12.8%)	0.184
Mortality in patients with absolute indication, *n* (%)			41 (33.1%)	25 (53.2%)	0.012
Good outcome in patients with relative indication, *n* (%)			22 (40.7%)	31 (42.5%)	0.541
Mortality in patients with relative indication, *n* (%)			17 (31.5%)	14 (19.2%)	0.177

*IQR* interquartile range, *LVO* large vessel occlusion, *mRS* modified Rankin Scale, *MT* mechanical thrombectomy, *n* number

^a^Comparing the group of patients with recanalized LVO to the group of patients with persisting LVO

### Regression analysis

Logistic regression showed age (OR 0.93, 95% CI 0.88–0.98), NIHSS (OR 0.80, 95% CI 0.67–0.95), IVT (OR 19.5, 95% CI 2.49–152.33), successful reperfusion (OR 94.34, 95% CI 3.80–2345.01), sICH (OR 0.02, 95% CI 0.00–0.51) and ground transportation (OR 7.46, 95% CI 1.43–38.97) to be independent predictors for good functional outcome (for details see Table 4).

**Table 4:** Logistic regression for the good functional outcome at 3 months

	OR	95% CI	*p* value
Age	0.927	0.875–0.982	0.010
Sex	1.140	0.249–5.210	0.866
NIHSS at admission	0.796	0.669–0.948	0.011
pmRS	0.379	0.129–1.110	0.077
CT ASPECTS	0.854	0.565–1.292	0.456
IVT treatment	19.491	2.494–152.330	0.005
mTICI 2b/3)	94.339	3.795–2345.006	0.006
Onset unknown	0.895	0.179–4.477	0.893
Time symptom onset to door at center	0.988	0.971–1.006	0.188
sICH-ECASS 4	0.015	0.000–0.510	0.020
Complications	0.364	0.065–2.023	0.248
Ground transportation	7.456	1.427–38.974	0.017

*ASPECTS* Alberta Stroke Program Early CT Score, *CI* confidence interval, *CT* computed tomography, *IVT* intravenous thrombolysis, *IQR* interquartile range, *LVO* large vessel occlusion, *MT* mechanical thrombectomy, *n* number, *mTICI* modified thrombolysis in cerebral infarction, *NIHSS* National Institute of Health Stroke Scale, *OR* odds ratio, *pmRS* premorbid modified Rankin Scale

Logistic regression for performing MT showed NIHSS (OR 1.01, 95% CI 1.05–1.15), ASPECTS (OR 1.26, 95% CI 1.08–1.48), and time window (OR 0.99, 95% CI 0.99–0.99), to be independent predictors (for details see Table 5).

**Table 5:** Logistic regression for performing mechanical thrombectomy after inter-hospital transfer

	OR	95% CI	*p* value
Age	0.982	0.958–1.007	0.161
Sex	1.652	0.869–3.140	0.125
NIHSS at admission	1.099	1.050–1.151	< 0.001
pmRS	0.854	0.634–1.150	0.299
CT ASPECTS	1.262	1.079–1.475	0.004
IVT treatment	0.903	0.466–1.750	0.763
Time symptom onset to door at center	0.994	0.991–0.998	0.002
Ground transportation	0.499	0.208–1.193	0.499

*ASPECTS* Alberta Stroke Program Early CT Score, *CI* confidence interval, *CT* computed tomography, *IVT* intravenous thrombolysis, *IQR* interquartile range, *LVO* large vessel occlusion, *MT* mechanical thrombectomy, *n* number, *NIHSS* National Institute of Health Stroke Scale, *OR* odds ratio, *pmRS* premorbid modified Rankin Scale

### Subgroup analysis of LVO-patients with absolute indication versus relative indication for MT

There were 124 patients (69.7%) with an absolute indication in the MT group and 47 (39.2%) in the no MT-group (*p* < 0.001). In these patients, a good outcome was numerically more frequent in the MT group (21.8% versus 12.8%, *p* = 0.184).

## Discussion

The main results of this analysis are the following.

First, only 12.0% out of 5722 NEVAS telemedicine consultations had the indication for inter-hospital transfer within the network to the University of Munich as a comprehensive stroke center. This low rate of patients who underwent inter-hospital transfer underlines that telemedicine stroke networks can avoid an unnecessary inter-hospital transfer. This is in good agreement with a recently published study from Catalonia [8]. A very recent study reports comparable rates of transferred but not treated patients [3].

Second, out of 350 patients suffering from LVO-stroke, 50.9% were finally treated with MT in our center. After inter-hospital transfer, 14.1% (*n* = 52) LVO were recanalized without a need for further intervention, in 50% after IVT-treatment. Main reasons for withholding MT in the group of patients with persisting LVO were imaging and clinical parameters that were suspected to presume non-successful treatment. Patients, who were finally treated with MT after inter-hospital transfer were characterized by shorter time window and a higher rate of IVT, higher ASPECTS, and higher NIHSS compared to non-treated patients. Regression analysis for performing MT showed that only stroke severity, ASPECTS and time window were independent predictors for performing MT. This observation of data in clinical routine reflects that the clinical decision-making process of MT was guided by the results of clinical trials. These trials described no upper limit for NIHSS and ASPECTS but less evidence for treatment in these cases. In our study, a shorter time window predicted good outcome and performing MT. MT-treated patients arrived in median 43.5 min earlier in our center than patients who were finally not treated (184.5 versus 228 min, *p* < 0.001), although onset to start IVT-treatment was 100.0 min in both MT and no-MT group (known in *n* = 176 patients with IVT in the external clinic). Regression analysis for good outcome showed that ground transportation was an independent predictor as median adjusted transportation time was 17.0 min shorter in ground transportation (*p* = 0.004). Although the pure flight time is likely to be shorter than that of ground transfer, the most likely reason for this delay is the lack of immediate availability of an aircraft. However, data on transportation time in the subgroup of IVT patients were only available in 50% of the patients. Thus, the interpretation of this observation is limited. In addition, prolonged time window was rarely a reason to withhold MT. However, we cannot exclude that in some patients delay in transportation for different reasons was a major reason for the withholding of MT.

One former study demonstrates that delay in transfer was associated with the withholding of MT treatment [1]. One other showed that established infarct, recanalized LVO and mild clinical symptoms were major causes of for no-treatment [1]. In contrast to another study, in our cohort age was not associated with MT [1, 2]. A very recent study reports comparable rates of transferred but not treated patients [3].

Third, strongest predictors for good outcome were treatment with IVT with an odds ratio of 19.4 and MT with an odds ratio of 94.3. That underlines the importance of recanalization therapies in LVO stroke patients within a telemedicine stroke network in real life. Although the overall outcome was different between the MT-group and the no MT-group, our sub analysis of patients with an absolute indication for MT according to clinical trials showed a good outcome twice as often in the MT-group than in the no-MT group, underlying the importance of MT for a good outcome.

Fourth, another important result of our study is that about 15% of LVO recanalized without intervention with or without IVT, which is in good agreement with recently reported rates of 10–20% of LVO recanalization under IVT [9, 10]. However, in the group of patients with persisting LVO we could not identify only one single predictor to select patients and determine whether MT will be performed or not.

Our study has the following strengths. For the best of our knowledge, this is the largest cohort of consecutive prospectively collected patients. In contrast to other studies, we had full access to the data on time intervals, periprocedural data and clinical outcome.

We are aware of some major limitations. We have no information about patients who were not transferred for different reasons that have not been described due to the design of our study. Thus, we cannot conclude about whether the decision to transfer or not was appropriate. In addition, our data do not allow to identify in detail why in the individual case a patient was treated with MT or not. With regard to this, our conclusions can only be generally.

In conclusion, we provide evidence that stroke care within a telemedicine network can be successfully organized as only a minority of patients has to be transferred to a comprehensive stroke center. All MT-eligible patients should be shipped with the fastest transportation modality as possible. However, no clear clinical parameters were found to predict in advance whether MT will be performed after transfer to the comprehensive stroke center or not.
